# Extraction of Traditional Chinese Medicine Entity: Design of a Novel Span-Level Named Entity Recognition Method With Distant Supervision

**DOI:** 10.2196/28219

**Published:** 2021-06-14

**Authors:** Qi Jia, Dezheng Zhang, Haifeng Xu, Yonghong Xie

**Affiliations:** 1 School of Computer and Communication Engineering University of Science and Technology Beijing Beijing China; 2 Beijing Key Laboratory of Knowledge Engineering for Materials Science Beijing China

**Keywords:** traditional Chinese medicine, named entity recognition, span level, distantly supervised

## Abstract

**Background:**

Traditional Chinese medicine (TCM) clinical records contain the symptoms of patients, diagnoses, and subsequent treatment of doctors. These records are important resources for research and analysis of TCM diagnosis knowledge. However, most of TCM clinical records are unstructured text. Therefore, a method to automatically extract medical entities from TCM clinical records is indispensable.

**Objective:**

Training a medical entity extracting model needs a large number of annotated corpus. The cost of annotated corpus is very high and there is a lack of gold-standard data sets for supervised learning methods. Therefore, we utilized distantly supervised named entity recognition (NER) to respond to the challenge.

**Methods:**

We propose a span-level distantly supervised NER approach to extract TCM medical entity. It utilizes the pretrained language model and a simple multilayer neural network as classifier to detect and classify entity. We also designed a negative sampling strategy for the span-level model. The strategy randomly selects negative samples in every epoch and filters the possible false-negative samples periodically. It reduces the bad influence from the false-negative samples.

**Results:**

We compare our methods with other baseline methods to illustrate the effectiveness of our method on a gold-standard data set. The F1 score of our method is 77.34 and it remarkably outperforms the other baselines.

**Conclusions:**

We developed a distantly supervised NER approach to extract medical entity from TCM clinical records. We estimated our approach on a TCM clinical record data set. Our experimental results indicate that the proposed approach achieves a better performance than other baselines.

## Introduction

### Background

As a complementary medicine with thousands of years history, traditional Chinese medicine (TCM) has received increasing attention and even played an important role in the fight against COVID-19 in China. TCM clinical records contain the symptoms and signs of patient and the diagnosis process of the doctor as unstructured text. These records represent a large number of valuable academic thoughts and clinical experience of TCM experts.

With information technology being applied to TCM modernization, it is essential to discover TCM diagnosis pattern through data mining [1]. While these studies rely on structured data, TCM clinical records are unstructured text. Besides, TCM clinical records are mostly recorded in ancient Chinese. The narrative is free style and difficult to understand for modern medical practitioners. The cost of manually structuring and maintaining free-text clinical records thus remains very expensive. Therefore, automatically extracting medical entities from TCM clinical records is an urgent need for research and analysis of TCM diagnosis knowledge.

So far, studies on medical entity extraction have mainly concentrated on modern medicine. The research on TCM medical entity extraction is still in early stages and faces more challenges. However, the text expression of TCM medical entity varies substantially and its boundaries are difficult to determine. For example, 牛黄解毒丸 (bezoar detoxicating tablet) is a prescription and includes a medicine 牛黄 (bezoar) in text. Because of these challenges, the cost of annotated corpus becomes very high; additionally, there is a lack of gold-standard data sets for supervised learning methods.

Distantly supervised named entity recognition (NER) is a good approach to deal with the situation where there is a lack of annotated corpus for training. It utilizes the domain entity dictionary and raw text to generate the silver-standard data set for training the NER model. In the TCM field, we can use existing knowledge resources to obtain the domain entity dictionary. However, the domain entity dictionary is always incomplete and cannot cover all entity names in practice. The diversity of TCM medical entities exacerbates this situation. In the silver-standard data set generated with distant supervision, each token that does not match the dictionary will be treated as a nonentity. As a result, it may introduce many false-negative samples and may have a bad influence on the performance of the NER model.

We therefore propose a span-level distantly supervised NER approach to extract TCM medical entity. Our key motivation is that although the entity mention could not be matched by the domain entity dictionary, the expression of the matched entity is correct. At this point, we treat the distantly supervised NER as a span detection task instead of a general sequence tagging task. We first design a simple classifier with a pretrained language model [2] to detect and type text span. It enumerates all possible text spans in a sentence as candidate entity mention and predicts the entity type of each text span independently. Compared with sequence tagging, the span-level method does not rely on the context token label in the sentence and reduces the influence of false-negative samples. The span-level entity extraction model needs to perform negative sampling as a nonentity that is not included in the domain entity dictionary. We then design a negative sampling strategy, which predicts the text span type periodically and evaluates the indeterminacy to filter the possible false-negative samples and reduce the influence on the model performance. We summarize the contribution as follows:

1. We propose a span-level distant supervised NER method to extract the TCM medical entity. It does not rely on the context token label in the sentence and mainly focuses on the feature of the entity span.
2. We also design a negative sampling strategy for our span-level entity extraction model. It filters the possible false-negative samples by measuring the indeterminacy of entity prediction to reduce the bad influence for the model training.
3. We estimate our approach on the TCM clinical record data set. Experimental results indicate that our approach achieves a better performance than other baselines.

### Related Work

In recent years, medical entity extraction has become a very popular topic. Early studies in this area mainly focused on the language lexicon pattern and domain dictionary. However, with the rapid development of deep learning, current mainstream research uses deep neural networks to extract medical entities by tagging text sequence. Habibi et al [3] presented a completely generic method based on long short-term memory (LSTM) neural network and statistical word embeddings. The method demonstrated improved recall, and performed better than traditional NER tools, thus confirming the effectiveness of this method over others. Cho and Lee [4] designed a contextual LSTM network with conditional random fields (CRFs), and proved that this method had significantly improved performance on biomedical NER tasks. Li et al [5] proposed an NER method called Bi-LSTM-Att-CRF that integrates the attention mechanism with a bidirection LSTM (Bi-LSTM) neural network to extract Chinese electronic medical records. This method not only captured more useful contextual information, but also introduced medical dictionaries and part-of-speech features to improve the performance of the model. While using the attention-Bi-LSTM-CRF model, Ji et al [6] used the entity auto-correct algorithm to rectify entities based on historical entity information which further improved the performance of the model. Wu et al [7] used the bidirectional encoder representations from transformers (BERT) [2] pretrained language model as a encoder to generate token embedding and incorporated it with several common deep learning models (eg, Bi-LSTM and Bi-LSTM-CRF).

The TCM medical entity extraction has gradually attracted the attention of scholars worldwide. Wang et al [8] used CRF to extract symptom entities from free-text clinical records. Wang et al [9] investigated supervised methods and verified their effectiveness in extracting TCM clinical records and focused on the problems related to symptom entity recognition. Wang et al [10] proposed a supervised method for syndrome segmentation.

However, all these methods relied on high-quality annotation data. In practice, the cost of this gold-standard data set is very high, and therefore, many studies have begun to study the distantly supervised NER method. Ren et al [11] proposed a novel relation phrase-based clustering framework while investigating entity recognition with distant supervision. Their framework uses some linguistic features such as part-of-speech. To use a small amount of labeled data for aspect term extraction, Giannakopoulos et al [12] introduced an architecture that achieves top-ranking performance for supervised aspect term extraction. Shang et al [13] designed a novel and effective neural model (AutoNER) with a new Tie or Break scheme. Through experiments, they proved the effectiveness of AutoNER when only using dictionaries with no additional human effort. Peng et al [14] proposed a novel positive-unlabeled (PU) learning algorithm to perform NER. The performance of this method was very dependent on the settings of important hyperparameters. Zhang et al [15] followed the corresponding annotation guidelines for clinical records of Chinese medicine and constructed a fine-grained entity annotation corpus. Zhang et al [16] proposed a novel back-labeling approach and integrated it into a tagging scheme, which improved the effectiveness and robustness of distantly supervised methods. These studies were, however, very much dependent on some a priori assumptions and external nature language process toolkits to denoise distantly supervised data set, and limit the task to sequence tagging.

## Methods

### Data

**Figure 1:**
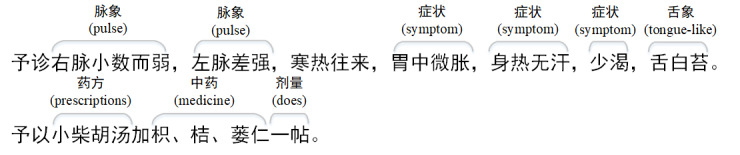
A TCM clinical record extraction example.

Distantly supervised NER needs a domain entity dictionary and raw text as the basic data. Therefore, first, we define the TCM medical entity type including 症状 (symptom), 脉象 (pulse), 舌象 (tongue like), 药方 (prescriptions), 中药 (medicine), and 剂量 (dose). We then obtain the domain dictionary from the TCM knowledge graph [17]. The dictionary includes 18,688 symptom entities, 34,594 Chinese medicine entities, 39,640 prescriptions entities, 304 dose entities, 4915 tongue entities, and 5800 pulse entities. We used a book entitled 《中华历代名医医案》 [18] as the raw text. It was compiled by the expert team of Professor Lu Zhaolin, Beijing University of Chinese Medicine, and has been published by the Beijing Science and Technology Publishing House. The book has collected more than 18,000 TCM cases and contains more than 8 million words. Each case introduces the patient’s illness involving the symptoms, pulse, and tongue like, and the process of seeking medical treatment. It also introduces the doctor’s diagnosis of the patient’s condition and a description of how to treat along with the medical prescription (in Chinese) and the corresponding dose of the prescribed medicine. Figure 1 shows an extracted sample record.

We used the maximum matching algorithm [19] to label back the medical entity. Specifically, there are conflicts in the dictionary such as 牛黄解毒丸 (bezoar detoxicating tablet) and 牛黄 (bezoar). For this case, we selected the longest text to match. We filtered out the sentences whose length was less than 15 tokens and did not match their entity in dictionary to generate the silver-standard data set.

### Span-Level NER Model

#### Overview

In this section, we explicate our span-level NER model in detail. Instead of the general sequence tagging model to extract name entity, the proposed model treats the task as a text-span classification and takes an arbitrary continuous text span as candidate input. For a given sentence *s* = [*t*_1_, *t*_2_, …, *t_N_*] of *n* token, there are *n*(*n* + 1)/2 possible text span. A text span is defined as span = [*t_i_*, …, *t_i+k_*], where 1 < *i* < *N* and *k* ≥ 0.

**Figure 2:**
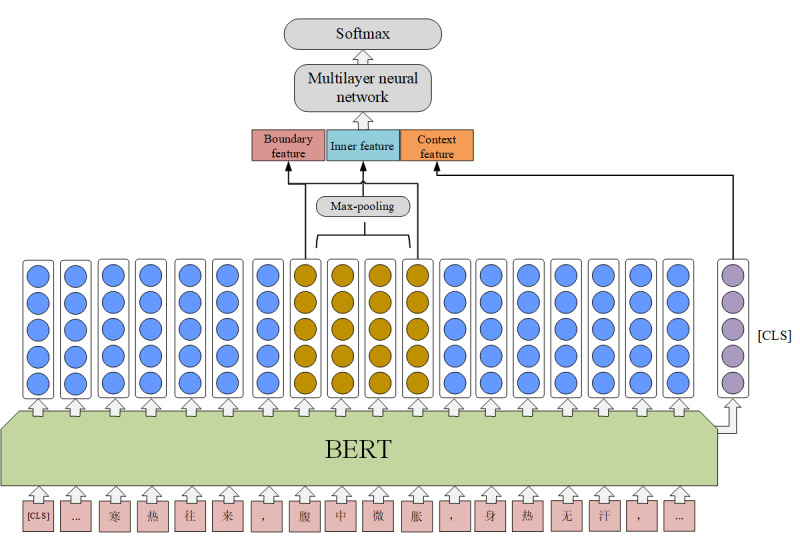
The span-level named entity extraction model. BERT: bidirectional encoder representations from transformers.

We designed a simple classifier to detect the entity type of text span (Figure 2). It utilizes BERT pretrained language model as text feature encoder and obtains the token embedding representation of text span. The BERT transforms the input token *t_i_* to an embedding vector *e_i_* with a length of 768. The representation of text span is a 2D tensor [*e_i_*, …, *e_i_*_+_*_k_*], where *k* is the length of span. The BERT pretrained language model keeps fine-tuning during training. Then, we model span representation as described in the following subsections.

#### Span Inner Feature

We combine the token embedding of the text span with max-pooling to represent the inner feature of span.

*R*_inner_(span)=maxpooling ([*e_i_*, …, *e_i_*_+_*_k_*]) (1)

#### Span Boundary Feature

For TCM medical entity, prefixes and suffixes have strong indications for the type of entity. We concatenate the head and tail token embedding as the boundary representation of span.

R_boundary_(span)=[e*_i_*;e*_i_*_+_*_k_*] (2)

We concatenate the span inner feature and the span boundary feature. In addition, we concatenate the representation of [CLS] in the BERT as the global sentence representation. The final span presentation is as follows:

*R*_span_=[*R*_inner_;*R*_boundary_;CLS] (3)

We feed the span representation to a multilayer neural network (2 layers in our model) and a softmax classifier which yields a posterior for each entity type.

*R*^s^=*f*_multi_(*W*·*R*_span_+*b*) (4)

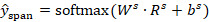
 (5)

### Negative Sample During Training

The most important problem in distantly supervised NER is the false-negative samples. During the training phase, the proposed span-level method needs to select nonentity text span as negative samples. We thus designed a negative sampling strategy on the silver-standard data set. Instead of using all the possible negative samples for training, the strategy randomly selects a number of negative samples in each epoch. It reduces the bad influence in the training phase from false-negative samples through label smoothing. Meanwhile, the model predicts the silver data set for several epochs periodically. We measure the indeterminacy of the prediction results by information entropy. According to the indeterminacy, we design a negative sample filter mechanism, which filters the possible false-negative samples in the next training period.

In each epoch, we randomly select the nonentity text span of the silver data set as negative samples. Because there may be entities in these negative samples, we use label smoothing to assign probability of entity types to negative samples:


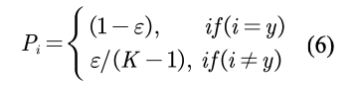


where *K* is the number of class and ε is a hyperparameter. During the training, we predict the data set for several epochs periodically. For each sentence *s_i_* in the data set, we predict the entity-type probability of each text span to obtain prediction result *R_i_*. We measure the indeterminacy with information entropy. The greater the information entropy, the greater the indeterminacy of the prediction of the text span.

**Figure 3:**
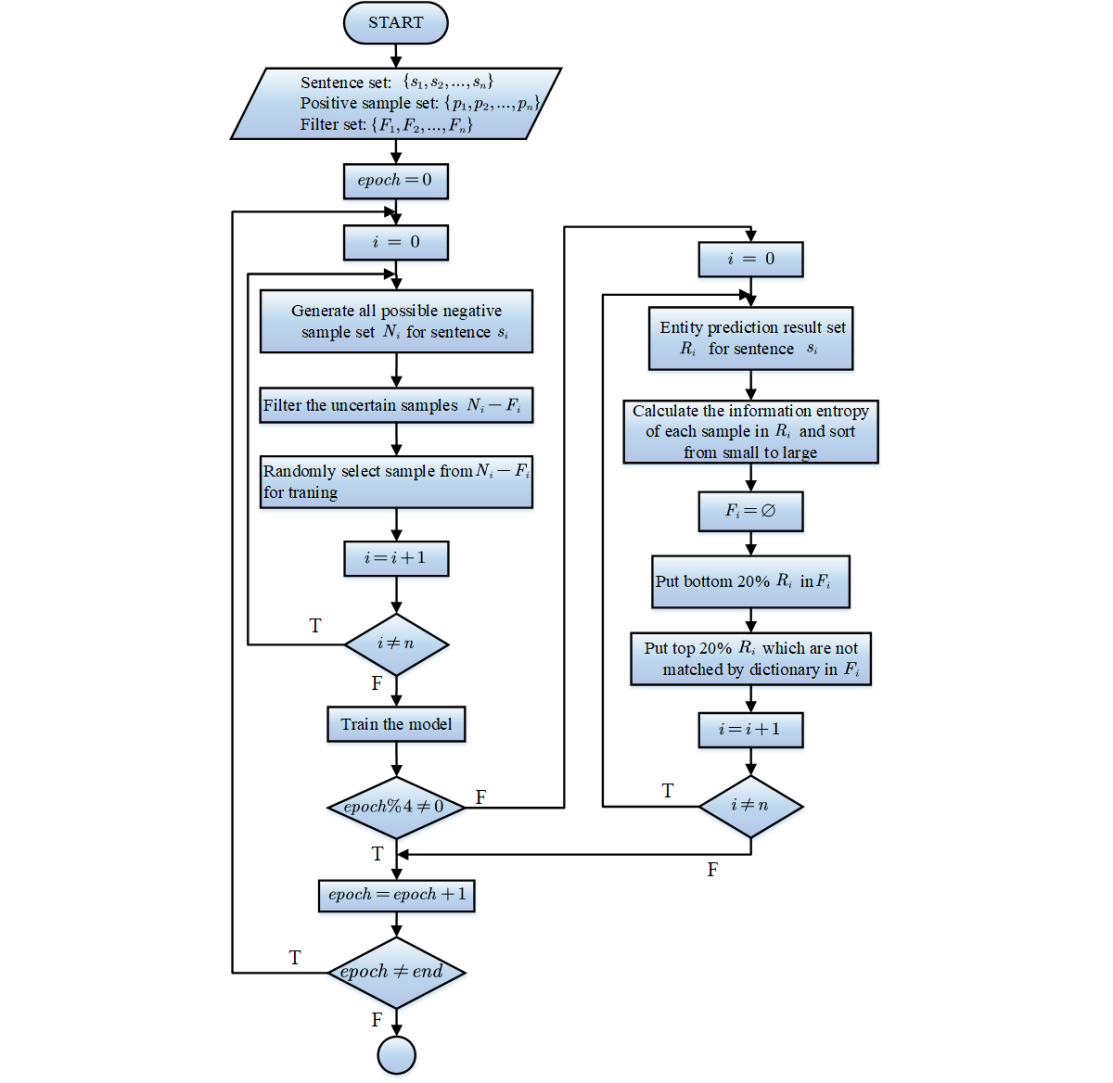
The flow diagram of the negative sampling strategy.

Then we sort *R_i_* (ie, the prediction result from small to large) according to the indeterminacy and maintain a corresponding negative-sample filtering set *F_i_* for each sentence *s_i_*. We put the bottom 20% sample and the top 20% entity sample, which are not matched by the dictionary of *R_i_*, in *F_i_*. The samples in *F_i_* are possible false-negative samples. These possible false-negative samples influence the model performance, and so we filter these samples in the model training phase. In the next training period, the samples in *F_i_* would not be selected in negative sampling. The flow diagram of the strategy is shown in Figure 3.

The purpose of this strategy is to avoid the influence of possible false-negative samples on the model. Filtering these samples out helps reduce the influence of incorrect labeling types on training.

## Results

### Data Set

The silver data set contains 203,485 tokens (10,083 sentences). In order to verify the effectiveness of our approach, we manually annotated 5000 sentences as the test set. It includes about 100,000 tokens with label. We also use the dictionary to label back the entity in the test set. The entity distribution is shown in Table 1. In particular, both the test set and the silver data set are from the same book, and there is no cross between them.

**Table 1:** The entity distribution on the test set in the dictionary and manual way.

Entity type	Dictionary	Manual
Symptom	5031	6362
Medicine	4854	8187
Prescriptions	450	545
Dose	6078	9276
Tongue-like	322	631
Pulse	668	972

### Experiment Set

During the process of model training, we used AdamW as the optimizer, and set the learning rate to 0.001 and the learning rate decay to 0.95. For the negative sampling strategy, we set the label smoothing *ε* to 0.85, the training epoch to 20, and the period to 4, and randomly select 100 negative samples for a sentence in an epoch. In this case, the filter set of negative sampling will update 4 times in training. According to the max text length in the domain entity dictionary, we limit the max length of span to 12 tokens.

The standard precision (P), recall (R), and F1 score (F1) on the test set are used as evaluation metrics. We compare the following baseline method to illustrate the effectiveness of our method.

Distant-LSTM-CRF [12] introduced domain-term mining and linguistic features such as dependency tree to generate the silver-standard data set. It used the sequence-tagging LSTM-CRF method to recognize entity by training on the silver-standard data set.

AdaPU [14] treated the distantly supervised NER as a positive unlabeled problem. This method generated a silver-standard data set with the maximum matching algorithm and trained LSTM-CRF models separately for each entity type. It designed a loss function depending on 2 hyperparameters: the ratio of entity words and the class weight for the positive class.

The novel label-back AutoNER [16] combined a domain term dictionary generated by AutoPhrase [20]. It designed a label-back strategy according to some prior assumptions to generate the silver-standard data set. The model masked the nonentity term during training to skip the parameter update.

BERT-CRF [2] is a popular supervised method. It is a sequence-tagging method and utilizes the BERT pretrained language model as encoder and CRF as sequence decoder. We used the silver data set in accordance with the proposed method as the training data set.

### Evaluation

The performance on the test set of the different methods is presented in Table 2. According to the results, the F1 score of our method is 77.34 and it remarkably outperforms the best baseline (novel label-back AutoNER) by an improvement of 8.16 in the F1 score. This indicates the effectiveness of the proposed method.

Compared with other baseline methods, our method shows substantial improvement in recall and makes a balance between the precision and recall. As a supervised method, BERT-CRF has a better performance than some distantly supervised methods on the silver-standard data set. This illustrates the effectiveness and robustness of the pretrained language model.

**Table 2:** Experiment results on the test set with comparison.

Method	Precision	Recall	F1 score
Distantly LSTM^a^-CRF^b^	74.03	31.59	53.93
AdaPU	70.15	60.87	65.18
Novel label-back AutoNER	73.06	66.75	69.18
BERT^c^-CRF	75.62	58.73	66.15
Our method	78.28	76.52	77.34

^a^LSTM: long short-term memory.

^b^CRF: conditional random field.

^c^BERT: bidirectional encoder representations from transformers.

## Discussion

### Principal Findings

In this section, we discuss the influence of negative sampling strategy on performance and the hyperparameter setting. We analyzed the effect of negative sampling strategy through an ablation study. Steps involved in the ablation study are as follows: (1) the random negative sampling is maintained, but the false-negative sample filter is removed; (2) the bottom sampling in the prediction result *R_i_* is removed; (3) the top sampling in the prediction result *R_i_* is removed; and (4) the label smoothing is removed. The result is presented in Table 3.

Based on the result, the F1 score is reduced by 4.60 without the false-negative sample filter. The false-negative sample filter mechanism avoids the influence of error samples on the model. It proves the validity of the false-negative sample filter mechanism. We also discuss the filter range. The bottom filter affects the performance more than the top filter, which illustrates that samples with larger indeterminacy influence the performance more.

Meanwhile, we also analyzed the influence of the span feature with the ablation study. The span representation includes the inner feature and the boundary feature. The result is shown in Table 4. Both the inner feature and the boundary feature will impact the model performance. In comparison with the boundary feature, the inner feature shows more obvious impact.

Moreover, we discuss the hyperparameters of the negative sampling including the number of random negative samples and the ratio of the false-negative sample filter. The number of random negative samples is set as 50, 100, 150, and 200, and the ratio of false-negative sample filter is set as 10%, 15%, 20%, and 25%. The result is presented in Tables 5 and 6. The obtained result indicates that the hyperparameters need to be set to appropriate values. We also notice that the ratio of the false-negative sample filter has a greater influence on the performance, and we consider this phenomenon to be caused by the coverage of the domain entity dictionary.

However, our study still has some limitations and could be improved. During the process of training and prediction, the method needs to enumerate all possible text spans in the sentence. This step affects the efficiency of the method. We consider introducing a toolkit such as word segmentation to improve the efficiency; otherwise we only consider the nonentity sampling and ignore the possible entity. For a specific domain, an entity name in dictionary has strong uniqueness and is not prone to ambiguity. However, in the open domain, the ambiguity for an entity name is common. Our method did not consider the false-positive samples because of the ambiguity. We intend to introduce some sampling strategies (eg, AdaSampling) and some self-supervised methods to solve the problem in future work.

**Table 3:** Experimental result without the false-negative sample filter.

Method	Precision	Recall	F1 score
Without the false-negative sample filter	75.15	70.48	72.74
Without bottom	75.93	73.25	74.57
Without top	76.86	75.75	76.30

**Table 4:** Experimental result without the false-negative sample filter.

Method	Precision	Recall	F1 score
Inner feature only	77.03	75.71	76.36
Boundary feature only	76.12	74.92	75.52

**Table 5:** Experimental results on different numbers of random negative samples.

Number of random negative samples	Precision	Recall	F1 score
50	77.24	75.17	76.19
100	78.28	76.52	77.34
150	78.42	75.25	76.80
200	79.56	72.91	76.09

**Table 6:** Experimental results on different ratios of the false-negative sample filter.

Number of random negative samples	Precision	Recall	F1 score
10%	76.25	71.34	73.71
15%	77.73	75.84	76.77
20%	78.28	76.52	77.34
25%	75.04.	75.28	75.15

### Conclusions

In this paper, we illustrated a distantly supervised NER approach to extract medical entity from TCM clinical records. Different from general sequence tagging, we propose a span-level model to detect and classify entity. It utilizes the pretrained language model as the text feature extractor, and constructs the span representation containing inner, boundary, and context feature. The model uses a multilayer neural network and softmax as classifier for the span representation. We designed a negative sampling strategy for the span-level model. The strategy randomly selects negative samples in every epoch and filters the possible false-negative sample periodically. We evaluated the effectiveness of our method by comparing it with other baselines. Meanwhile, we also discussed the influence of different parts of negative sampling strategy on performance. In the future, we intend to extend our method to a wider range of fields and study its generalization. We also will optimize the negative sampling strategy to improve the ability to filter the false-negative samples.

## Abbreviations

CRF: conditional random fields
LSTM: long short-term memory
NER: named entity recognition
POS: part-of-speech
TCM: traditional Chinese medicine
